# Suitability of existing *Musa* morphological descriptors to characterize East African highland ‘matooke’ bananas

**DOI:** 10.1007/s10722-017-0562-9

**Published:** 2017-09-18

**Authors:** Michael Batte, Alex Mukiibi, Rony Swennen, Brigitte Uwimana, Luis Pocasangre, Helena Persson Hovmalm, Mulatu Geleta, Rodomiro Ortiz

**Affiliations:** 1 International Institute of Tropical Agriculture (IITA), P.O. Box 7878, Kampala, Uganda; 2 Swedish University of Agricultural Sciences (SLU), Sundsvágen 10, Box 101, 23053 Alnarp, Sweden; 3 EARTH University, San José 4442-1000, Costa Rica; 4 International Institute of Tropical Agriculture (IITA), C/o The Nelson Mandela African Institution for Science and Technology (NM-AIST), P.O. Box 447, Arusha, Tanzania; 5 Laboratory of Tropical Crop Improvement, Katholieke Universiteite Leuven (KUL), Willem De Croylaan 42, Bus 2455, 3001 Leuven, Belgium; 6 Bioversity International, Willem De Croylaan 42, 3001 Heverlee, Belgium

**Keywords:** Cultivar, Descriptors, East African highland bananas, Hybridization, Matooke

## Abstract

Morphological traits are commonly used for characterizing plant genetic resources. Germplasm characterization should be based on distinctly identifiable, stable and heritable traits that are expressed consistently and are easy to distinguish by the human eye. Characterization and documentation of a representative sample of East African highland bananas (Lujugira–Mutika subgroup) was carried out following an internationally accepted standard protocol for bananas. Eleven cultivars were characterized using an existing set of minimum descriptors (31 qualitative and quantitative traits) with the aim of determining stable descriptors and the ability of these descriptors to distinguish among East African highland banana cultivars. There was variation in stability of these descriptors within cultivars and across the 11 cultivars. Only 10 (32%) out of 31 descriptors studied were stable in the 11 cultivars. However, they had similar scores and therefore are not suitable to distinguish between cultivars within this group. Nonetheless, these 10 descriptors may be useful for distinguishing the East African highland bananas as a group from other groups of bananas. A few descriptors were unique to the cultivar ‘Tereza’ and may be used to distinguish this cultivar from other ‘matooke’ cultivars. None of the quantitative descriptors were stable.

## Introduction

Bananas (*Musa* spp. Linnaeus) can be divided into edible cultivars and non-edible wild species. The edible bananas include dessert, cooking and beer making cultivars, which mostly originated from Southeast Asia (Perrier et al. 2009). Their ancestors are *Musa acuminata* Colla denoted as AA and *Musa balbisiana* Colla (BB). The natural hybridization between and within *M. acuminata* and *M. balbisiana* resulted in several cultivars with different genomes and ploidy levels (Hippolyte et al. 2012). The possible genomic groups for bananas include; AA, BB, AB, AAA, AAB, ABB AABB, AAAB and ABBB (Creste et al. 2003).

The East African Highland bananas (*Musa* AAA group) also referred to as EAHB, is an endemic group of bananas found in the Great Lakes region (Uganda National Council for Science and Technology 2007). They are grown at altitudes between 900 and 2000 m above sea level, and are mainly found in Burundi, Kenya, Rwanda, Tanzania, and Uganda, plus in some areas of Cameroon and the Democratic Republic of Congo. The cultivars within the group require an average of 2000-2500 mm of rain evenly distributed throughout the year (Uganda National Council for Science and Technology 2007) as they are very drought susceptible (Kissel et al. 2015, 2016). The EAHB are placed in the Lujugira–Mutika subgroup, which has been further divided into the five clone sets Mbidde, Musakala, Nakabululu, Nakitembe and Nfuuka (Karamura 1998; Pickersgill and Karamura 1999). Each clone set is composed of a number of cultivars that serve different functions such as beer making (as Mbidde), or as being eaten as cooked food or dessert (all others), depending on the region where they are grown.

In Uganda, cooking banana cultivars are locally known as ‘matooke’ and serve as staple food to a large part of the population. Uganda produces over 8 million tons of ‘matooke’ bananas annually, which makes it the second largest banana producer in the world. The daily per capita consumption of ‘matooke’ in Uganda is 0.7 kg (ABSPII 2013), making it the most important food and cash crop for small-scale farmers in this country. Banana production in Uganda has, however, declined over the past two decades due to production constraints such as attacks by black Sigatoka, parasitic nematodes, bacterial wilt and banana weevil, and problems related to soil fertility and inadequate moisture during drought (Swennen et al. 2013). Banana breeding carried out by the International Institute of Tropical Agriculture (IITA) and the National Agricultural Research Organization (NARO) targets constraints related to pests and diseases (Tushemereirwe et al. 2015). Banana crossbreeding starts with the hybridization of EAHB with wild or improved diploids which have resistance to banana diseases and pests, to generate banana clones showing host plant resistance to biotic and abiotic stresses, short cycle and height, high yield and quality (Ortiz and Swennen 2014). To facilitate the access and use in *Musa* breeding, appropriate conservation, characterization and evaluation of genetic variation in the matooke banana cultigen pool is required.

Based on the Global Conservation Strategy for *Musa* spp, the Taxonomy Advisory Group (2010) agreed on a list of the minimum (32) set of descriptors for characterization and documentation of bananas. These banana descriptors allow discrimination between different cultivars in the field, in addition to monitoring morphological attributes that are highly heritable (Daniells et al. 2001). To standardize data recording, plants at the right developmental stage, i.e., when plants are green ripe or having a bunch rachis with 45 cm length, are selected for description (Channeliére et al. 2011). However, little is known about the stability of the selected descriptors in *Musa*. In the present study, characterization of a sample of EAHB belonging to two clone sets was carried out with the objective of identifying stable descriptors that could be used for conservation purposes, to distinguish cultivars in germplasm collections and also for breeding purposes, to select breeding materials, and to describe new cultivars developed by the breeding program.

## Materials and methods

Eleven female fertile East African Highland banana cultivars from two different clone sets (Table 1) were planted at the International Institute of Tropical Agriculture (IITA) in Namulonge/Sendusu, Uganda (00°31′47″N and 32°36′9″E at an elevation of 1167 m above sea level). The climate at this station fluctuates between dry and wet periods with an average temperature of 22 °C and average annual rainfall of 1264 mm (Nsubuga et al. 2011). A minimum of 5 plants and a maximum of 20 plants per cultivar within the same location were evaluated between September and December 2014 with *Musa* descriptors from the minimum descriptor list (Taxonomic Advisory Group 2010). For uniformity, the evaluation was done on plants having a bunch rachis of at least 60 nodes or a rachis of approximately 45 cm in length (Channeliére et al. 2011).

**Table 1:** Female fertile East African highland banana ‘matooke’ cultivars used in this study

Clone set	Nakabululu	Nfuuka
Cultivar	Kazirakwe	Entukura
	Nakasabira	Enyeru
	Nakayonga	Enzirabahima
	Nakyetengu	Kabucuragye
		Namwezi
		Nfuuka
		Tereza

Thirty-one descriptors were used to record the morpho-taxonomic characters on the 11 ‘matooke’ cultivars. Twenty-eight of the descriptors were qualitative, while three were quantitative. The quantitative descriptors were: fruit length (of the middle fruit of the third hand), number of hands per bunch and number of fruits on mid hand of the bunch (Tables 2, 3). The qualitative descriptors were: sap colour, edge of petiole margin, colour of cigar leaf dorsal surface, bract behaviour before falling, lobe colour of compound tepal, pseudostem height, predominant underlying colour of pseudostem, blotches at the petiole base, petiole canal leaf III, petiole margins, petiole margins colour, bunch position, bunch shape, rachis position, rachis appearance, male bud shape, bract apex shape, bract imbrication, colour of the bract external face, colour of bract internal face, compound tepal basic colour, anther colour, dominant colour of male flower, fruit shape, fruit apex, remains of flower relicts at fruit apex, fruit pedicel length and fusion of pedicels (Tables 4, 5). Size of male bud at harvest, which is supposed to be the 32nd descriptor according to the minimum descriptor list was not used in this study because the male buds were removed from plants before harvest to control the spread of banana bacterial wilt (Kubiriba and Tushemereirwe 2014). The descriptors related to color were examined using standard color charts developed by the Taxonomy Advisory Group (2010). All descriptor characters were recorded using scores ranging from 1 to 10, in a categorical manner, except the three quantitative descriptors which were measured and recorded directly.

**Table 2:** Fruit and bunch quantitative traits (mean ± SD) of eleven East African highland banana ‘matooke’ cultivars

Clone	Cultivar	Fruit length (cm)	Number of hands per bunch	Number of fruits on mid hand of bunch
Nakabululu	Kazirakwe	13.72 ± 1.48	5.72 ± 0.96	13.39 ± 1.65
	Nakasabira	12.62 ± 1.42	5.45 ± 1.10	12.45 ± 1.50
	Nakayonga	13.77 ± 1.49	6.33 ± 1.07	14.42 ± 1.56
	Nakyetengu	16.74 ± 1.44	5.80 ± 0.84	14.20 ± 2.87
Nfuuka	Entukura	14.76 ± 1.15	5.55 ± 0.69	12.82 ± 1.83
	Enyeru	14.39 ± 1.77	5.13 ± 1.02	13.94 ± 2.35
	Enzirabahima	15.24 ± 2.07	5.00 ± 1.21	13.33 ± 2.02
	Kabucuragye	17.73 ± 2.29	8.20 ± 1.69	17.10 ± 3.63
	Namwezi	12.73 ± 1.30	4.47 ± 0.84	11.79 ± 1.90
	Nfuuka	15.34 ± 0.94	6.25 ± 1.16	15.63 ± 2.72
	Tereza	17.09 ± 1.17	8.31 ± 1.40	17.75 ± 3.66

**Table 3:** One-way analysis of variance for quantitative fruit and bunch traits in eleven East African highland banana ‘matooke’ cultivars

Source	DF^z^	SS	MS	F_c_	*P* > F_c_
*Fruit length*					
Cultivar	10	396.91	39.691	16.845	<2.2e–16***
Residuals	136	320.46	2.356		
*Number of hands per bunch*					
Cultivar	10	213.24	21.3241	17.285	<2.2e–16***
Residuals	136	167.78	1.2337		
*Number of fruits on mid hand of the bunch*					
Cultivar	10	512.77	51.277	9.3342	1.137e–11***
Residuals	136	747.12	5.494		

*DF^z^* degrees of freedom, *SS* sum of squares, *MS* mean squares, *F_c_* F calculated

‘***’, ‘**’ and ‘*’ indicate that the source of variation was significant at *P* ≥ 0.001, 0.01 and 0.05, respectively

**Table 4:** Probability for binomial test of 28 categorical descriptors with null hypothesis *P* = 0.5 versus alternative hypothesis P>0.5 for 11 banana cultivars

Descriptor	Nakabululu clone set				Nfuuka clone set						
	Kazirakwe	Nakasabira	Nakayonga	Nakyetengu	Entukura	Enyeru	Enzirabahima	Kabucuragye	Namwezi	Nfuuka	Tereza
Sap colour	0.00000381***	0.00000095***	0.00024414***	0.03125*	0.00048828***	0.00001525***	0.00024414***	0.00097656***	0.00000190***	0.00390625**	0.00001525***
Edge of petiole margin	0.00000381***	0.00000095***	0.00024414***	0.03125*	0.00048828***	0.00001525***	0.00024414***	0.00097656***	0.00000190***	0.00390625**	0.00001525***
Colour of cigar leaf dorsal surface	0.00000381***	0.00000095***	0.00024414***	0.03125*	0.00048828***	0.00001525***	0.00024414***	0.00097656***	0.00000190***	0.00390625**	0.00001525***
Bract behaviour before falling	0.00000381***	0.00000095***	0.00024414***	0.03125*	0.00048828***	0.00001525***	0.00024414***	0.00097656***	0.00000190***	0.00390625**	0.00001525***
Lobe colour of compound tepal	0.00000381***	0.00000095***	0.00024414***	0.03125*	0.00048828***	0.00001525***	0.00024414***	0.00097656***	0.00000190***	0.00390625**	0.00001525***
Pseudostem height (m)	0.1189423NS	0.05765915NS	0.07299805NS	0.03125*	0.2744141NS	0.4018097NS	0.387207NS	0.01074219*	0.1796417NS	0.03515625*	0.00001525***
Predominant underlying colour of pseudostem	0.00000381***	0.00000095***	0.387207NS	0.031258*	0.00048828***	0.00001525***	0.00317382**	0.00097656***	0.00036430***	0.00390625**	0.00001525***
Blotches at the petiole base	0.11894226NS	0.00020122***	0.00024414***	0.5NS	0.27441406NS	0.22724914NS	0.19384765NS	0.00097656***	0.00960540**	0.14453125NS	0.01063537*
Petiole canal leaf III	0.00376892**	0.94234085NS	0.01928710*	0.1875NS	0.72558593NS	0.22724914NS	0.38720703NS	0.00097656***	0.32380294NS	0.00390625**	0.03840637*
Petiole margins	0.00000381***	0.00000095***	0.1938477NS	0.03125*	0.00585937**	0.00001525***	0.00024414***	0.00097656***	0.00003814***	0.00390625**	0.00001525***
Petiole margins colour	0.04812622*	0.00002002***	0.00317382**	0.03125*	0.00048828***	0.00001525***	0.1938477NS	0.00097656***	0.00000190***	0.00390625**	0.00001525***
Bunch position	0.24034118NS	0.13158798NS	0.61279296NS	0.8125NS	0.27441406NS	0.40180969NS	0.19384765NS	0.0546875NS	0.00221252**	0.85546875NS	0.77275085NS
Bunch shape	0.01544189*	0.4119015NS	0.01928711*	0.5NS	0.00585937**	0.00001525***	0.1938477NS	0.3769531NS	0.08353424NS	0.3632813NS	0.01063538*
Rachis position	0.59273529NS	0.00590896**	0.61279296NS	0.8125NS	0.88671875NS	0.59819030NS	0.80615234NS	0.0546875NS	0.00221252**	0.85546875NS	0.40180969NS
Rachis appearance	0.2403412NS	0.00000095***	0.00024414***	0.03125*	0.00048828***	0.00001525***	0.00024414***	0.0546875NS	0.00000190***	0.00390625**	0.00001525***
Male bud shape	0.00000381***	0.00000095***	0.01928711*	0.03125*	0.2744141NS	0.00025939***	0.1938477NS	0.01074219*	0.00000190***	0.1445313NS	0.03840637NS
Bract apex shape	0.00000381***	0.00000095***	0.00024414***	0.1875NS	0.2744141NS	0.1050568NS	0.1938477NS	0.01074219*	0.5NS	0.1445313NS	0.00001525***
Bract imbrication	0.00000381***	0.00002002***	0.00024414***	0.03125*	0.00048828***	0.00001525***	0.00024414***	0.00097656***	0.00000190***	0.00390625**	0.00001525***
Colour of the bract external face	0.00000381***	0.00000095***	0.00024414***	0.03125*	0.00048828***	0.00001525***	0.1938477NS	0.00097656***	0.00000190***	0.00390625**	0.00001525***
Colour of bract internal face	0.00000381***	0.00000095***	0.00024414***	0.03125*	0.1132812NS	0.00025939***	0.07299805NS	0.00097656***	0.00000190***	0.00390625**	0.00001525***
Compound tepal basic colour	0.00000381***	0.00000095***	0.00024414***	0.03125*	0.00048828***	0.00025939***	0.00024414***	0.00097656***	0.00000190***	0.00390625**	0.00001525***
Anther colour	0.00000381***	0.00000095***	0.00024414***	0.03125*	0.00048828***	0.00025939***	0.00024414***	0.00097656***	0.00000190***	0.00390625**	0.00001525***
Dominant colour of male flower	0.00000381***	0.00000095***	0.00024414***	0.03125*	0.00048828***	0.00025939***	0.00024414***	0.00097656***	0.00000190***	0.00390625**	0.01063538*
Fruit shape	0.00007247***	0.00000095***	0.00317382**	0.03125*	0.00048828***	0.00025939***	0.00024414***	0.00097656***	0.00036430***	0.00390625**	0.00001525***
Fruit apex	0.40726470NS	0.05765914NS	0.19384765NS	0.5NS	0.03271484*	0.03840637*	0.07299804NS	0.01074218*	0.5NS	0.00390625**	0.00025939***
Remains of flower relicts at fruit apex	0.95187378NS	0.74827766NS	0.80615234NS	0.1875NS	0.88671875NS	0.03840637*	0.07299805NS	0.37695313NS	0.32380295NS	0.36328125NS	0.03840637*
Fruit pedicel length (mm)	0.11894226NS	0.05765914NS	0.07299804NS	0.5NS	0.00048828***	0.01063537*	0.00317382**	0.00097656***	0.00036430***	0.36328125NS	0.10505676NS
Fusion of pedicels	0.01544189*	0.4119015NS	0.01928711*	0.03125*	0.00048828***	0.00001525***	0.00024414***	0.00097656***	0.00000190***	0.00390625**	0.00001525***

(‘***’) highly stable descriptor, (‘**’) moderately stable descriptor, (‘*’) fairly stable descriptor and (NS) unstable descriptor

**Table 5:** Lower bounds of the one-sided confidence interval for 28 descriptors of 11 banana cultivars (The upper bound being 1)

Descriptor	Nakabululu clone set				Nfuuka clone set						
	Kazirakwe	Nakasabira	Nakayonga	Nakyetengu	Entukura	Enyeru	Enzirabahima	Kabucuragye	Namwezi	Nfuuka	Tereza
Sap colour	0.85	0.86	0.78	0.55	0.76	0.83	0.78	0.74	0.85	0.69	0.83
Edge of petiole margin	0.85	0.86	0.78	0.55	0.76	0.83	0.78	0.74	0.85	0.69	0.83
Colour of cigar leaf dorsal surface	0.85	0.86	0.78	0.55	0.76	0.83	0.78	0.74	0.85	0.69	0.83
Bract behaviour before falling	0.85	0.86	0.78	0.55	0.76	0.83	0.78	0.74	0.85	0.69	0.83
Lobe colour of compound tepal	0.85	0.86	0.78	0.55	0.76	0.83	0.78	0.74	0.85	0.69	0.83
Pseudostem height (m)	0.45	0.49	0.47	0.55	0.35	0.33	0.32	0.61	0.42	0.53	0.83
Predominant underlying colour of pseudostem	0.85	0.86	0.32	0.55	0.76	0.83	0.66	0.74	0.70	0.69	0.83
Blotches at the petiole base	0.45	0.72	0.78	0.19	0.35	0.39	0.39	0.74	0.58	0.40	0.58
Petiole canal leaf III	0.62	0.18	0.56	0.34	0.20	0.39	0.32	0.74	0.37	0.69	0.52
Petiole margins	0.85	0.86	0.39	0.55	0.64	0.83	0.78	0.74	0.77	0.69	0.83
Petiole margins colour	0.50	0.78	0.66	0.55	0.76	0.83	0.39	0.74	0.85	0.69	0.83
Bunch position	0.39	0.44	0.25	0.08	0.35	0.33	0.39	0.49	0.64	0.11	0.23
Bunch shape	0.56	0.35	0.56	0.19	0.64	0.83	0.39	0.30	0.47	0.29	0.58
Rachis position	0.29	0.60	0.25	0.08	0.14	0.28	0.18	0.49	0.64	0.11	0.33
Rachis appearance	0.39	0.86	0.78	0.55	0.76	0.83	0.78	0.49	0.85	0.69	0.83
Male bud shape	0.85	0.86	0.56	0.55	0.35	0.74	0.39	0.61	0.85	0.40	0.52
Bract apex shape	0.85	0.86	0.78	0.34	0.35	0.45	0.39	0.61	0.32	0.40	0.83
Bract imbrication	0.85	0.78	0.78	0.55	0.76	0.83	0.78	0.74	0.85	0.69	0.83
Colour of the bract external face	0.85	0.86	0.78	0.55	0.76	0.83	0.39	0.74	0.85	0.69	0.83
Colour of bract internal face	0.85	0.86	0.78	0.55	0.44	0.74	0.47	0.74	0.85	0.69	0.83
Compound tepal basic colour	0.85	0.86	0.78	0.55	0.76	0.74	0.78	0.74	0.85	0.69	0.83
Anther colour	0.85	0.86	0.78	0.55	0.76	0.74	0.78	0.74	0.85	0.69	0.83
Dominant colour of male flower	0.85	0.86	0.78	0.55	0.76	0.74	0.78	0.74	0.85	0.69	0.58
Fruit shape	0.76	0.86	0.66	0.55	0.76	0.74	0.78	0.74	0.70	0.69	0.83
Fruit apex	0.34	0.49	0.39	0.19	0.53	0.52	0.47	0.61	0.32	0.69	0.74
Remains of flower relicts at fruit apex	0.16	0.26	0.18	0.34	0.14	0.52	0.47	0.30	0.37	0.29	0.52
Fruit pedicel length (mm)	0.45	0.49	0.47	0.19	0.76	0.58	0.66	0.74	0.70	0.29	0.45
Fusion of pedicels	0.56	0.35	0.56	0.55	0.76	0.83	0.78	0.74	0.85	0.69	0.83

Data were analyzed using R-software version 3.2.0 (R Core Team 2015). Categorical variables were first converted to binary scale by calculating mode of the data set. The mode scores were given a value of 0 while the non-mode scores were given a value of 1. The data were then analyzed by binomial test at 95% confidence level, the null hypothesis being that “the probability of getting a mode score is equal to the probability of getting a non-mode score (*P* = 0.5)”, while the alternative hypothesis was “the probability of getting a mode score is greater than 0.5 (*P* > 0.5)”. One way lower class boundaries were also calculated to determine the location of the mode. The means and standard deviations for the quantitative data were calculated (Table 2). One-way analysis of variance was done for the quantitative data (Table 3).

The stable (monomorphic) descriptors identified in this study were used to compare the 11 ‘matooke’ cultivars with banana cultivars from other groups. Consequently, seven dessert (AAA) bananas (Table 6), five Asian cooking (ABB) banana cultivars (Table 7) and 15 East African Highland banana cultivars belonging to five clone sets (Table 8) were compared using the identified stable qualitative descriptors. The data for these three additional banana groups were obtained from *Musalogue*, which is an international catalogue for *Musa* germplasm (Daniells et al. 2001). The data were first converted to binary scale using the mode. The mode scores were given a value of 0 while the non-mode scores were given a value of 1. Then data were used to cluster the banana groups using Ward’s hierarchical agglomerative clustering method (Murtagh and Legendre 2014).

**Table 6:** Dessert bananas (AAA) characterized using 10 monomorphic descriptors. *Source:* Daniells et al. (2001)

Descriptor	Gros Michel	Highgate	Petite Naine	Grande Naine	Williams	Red Dacca	Ibota
Sap colour	Watery	Watery	Watery	Milky	Milky	Milky	Milky
Edge of petiole margin	-	-	-	-	-	-	-
Colour of cigar leaf dorsal surface	Green	Green	Green	Green	Green	Green	Green
Bract behavior before falling	Revolute (rolling)	Revolute (rolling)	Revolute (rolling)	Revolute (rolling)	Revolute (rolling)	Revolute (rolling)	Not revolute (not rolling)
Lobe colour of compound tepal	Yellow	Yellow	Yellow	Yellow	Yellow	Yellow	Yellow
Bract imbrication	No imbrication	Moderate imbrication	Moderate imbrication	Moderate imbrication	Moderate imbrication	No imbrication	Deep imbrication
Compound tepal basic	Cream	Cream	Cream	Cream	Cream	Cream	Cream
Anther colour	-	-	-	-	-	-	-
Dominant colour of male	-	-	-	-	-	-	-
Fruit shape	Straight in the distal part	Curved	Curved	Curved	Curved	Straight (or slightly curved)	Straight (or slightly curved)

**Table 7:** Asian cooking bananas (ABB) characterized using 10 monomorphic descriptors. *Source:* Daniells et al. (2001)

Descriptor	Ducasse	Monthan	Birbutia	Saba	Pelipita
Sap colour	Milky	Watery	Watery	Watery	-
Edge of petiole margin	-	-	-	-	-
Colour of cigar leaf dorsal surface	Green	Green	Green	Green	Green
Bract behavior before falling	Revolute (rolling)	Revolute (rolling)	Not revolute (not rolling)	Revolute (rolling)	Not revolute (not rolling)
Lobe colour of compound tepal	Yellow	Yellow	Yellow	Yellow	Orange
Bract imbrication	Moderate imbrication	Moderate imbrication	Moderate imbrication	Deep imbrication	Deep imbrication
Compound tepal basic colour	Cream	Cream	Cream	Cream	Cream
Anther colour	-	-	-	-	-
Dominant colour of male flower	-	-	-	-	-
Fruit shape	Straight in the distal part	Straight (or slightly curved)	Straight (or slightly curved)	Straight (or slightly curved)	Curved

**Table 8:** East African highland banana cultivars belonging to 5 clone sets characterized using 10 monomorphic descriptors. *Source:* Daniells et al. (2001)

Descriptor	Musakala clone set			Nakabululu clone set			Nakitembe clone set		
	Mpologoma	Muvubo	Namunwe	Nakabululu	Butobe	Entente	Mbwazirume	Nakitembe	Namaliga
Sap colour	Milky	Milky	Milky	Milky	Milky	Milky	Milky	Milky	Milky
Edge of petiole margin	-	-	-	-	-		-	-	-
Colour of cigar leaf dorsal surface	Green	Green	Green	Green	Green	Green	Green	Green	Green
Bract behavior before falling	Revolute	Revolute	Revolute	Revolute	-	Revolute	Revolute	Revolute	Revolute
Lobe colour of compound tepal	Yellow	Yellow	Yellow	Yellow	Yellow	Yellow	Yellow	Yellow	Yellow
Bract imbrication	-	-	-	-	-	-	No imbrication	No imbrication	No imbrication
Compound tepal basic colour	Cream	Cream	Cream	Cream	Cream	Cream	Cream	Cream	Cream
Anther colour	-	-	-	-	-	-	-	-	-
Dominant colour of male flower	-	-	-	-	-	-	-	-	-
Fruit shape	Curved, slender	Curved, slender	Curved, slender	Straight or slightly curved	Straight or slightly curved	Straight or slightly curved	Straight or slightly curved	Straight in the distal part	Curved

Descriptor	Nfuuka clone set			Mbidde clone set		
	Nakibira	Nasuuna	Ntika	Nalukira	Kabula	Nsowe
Sap colour	Milky	Milky	Milky	Milky	Milky	Milky
Edge of petiole margin	-	-	-	-	-	-
Colour of cigar leaf dorsal surface	Green	Green	Green	Green	Green	Green
Bract behavior before falling	Revolute	Revolute	Revolute	Revolute	Revolute	Revolute
Lobe colour of compound tepal	Yellow	Yellow	Yellow	Yellow	Yellow	Yellow
Bract imbrication	-	-	-	-	-	-
Compound tepal basic colour	Cream	Cream	Cream	Cream	Cream	Cream
Anther colour	-	-	-	-	-	-
Dominant colour of male flower	-	-	-	-	-	-
Fruit shape	Straight or slightly curved	Straight or slightly curved	Straight or slightly curved	Straight or slightly curved	Straight or slightly curved	Straight or slightly curved

## Results

The variation for fruit length, number of hands per bunch and number of fruits on the mid hand of the bunch among the 11 female fertile East African highland bananas is given in Table 2. One-way analysis of variance indicated that the cultivars were significantly different for these traits (Table 3).

Within each cultivar, there was variation for stability of the qualitative descriptors used ranging from highly stable (‘***’), moderately stable (‘**’), fairly stable (‘*’) to unstable (NS) (Table 4). Ten descriptors of which six being flowering related, were stable across all the 11 ‘matooke’ cultivars (Table 4). These descriptors were: sap colour, edge of petiole margin, colour of cigar leaf dorsal surface, bract behaviour before falling, lobe colour of compound tepal, bract imbrication, compound tepal basic colour, anther colour, dominant colour of male flower and fruit shape. The stable descriptors stretched across the two clone sets and there was no set of stable descriptors observed in only one clone set. Only cultivar ‘Tereza’ had characters that were unique from all the others cultivars. These characters were colour of the bract external face and colour of bract internal face (Supplementary Figure S1).

The lower bounds of the mode scores for the qualitative descriptors varied from 0.08 (8%) to 0.86 (86%) across all the 11 cultivars (Table 5). All descriptors with *P* values having ‘***’, ‘**’ and ‘*’ levels of significance (Table 4) had their corresponding lower bounds higher than 0.5 (50%) (Table 5), while the descriptors with *P* values showing ‘NS’ had their lower bound values less than 0.5 (50%).

**Fig. 1:**
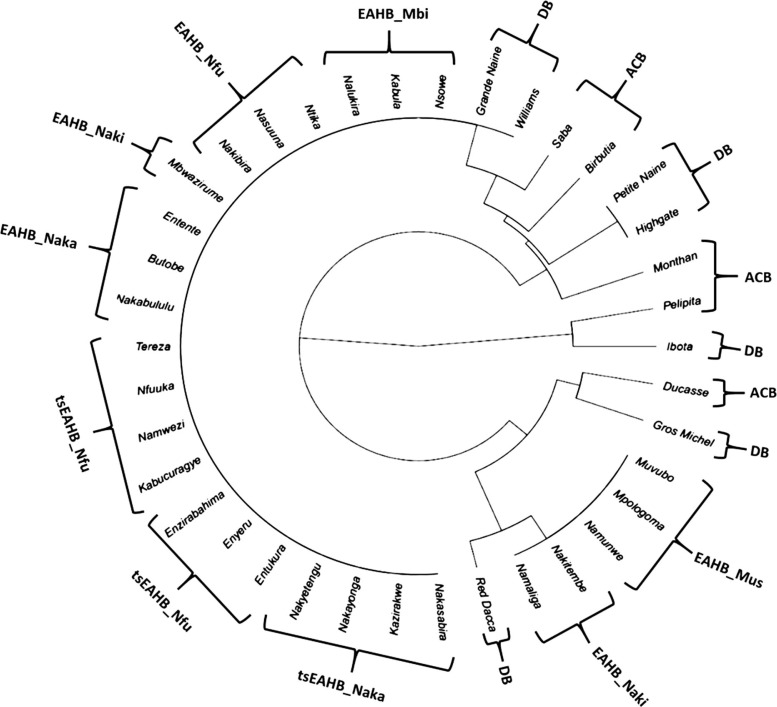
A cladogram showing clustering of 11 female fertile East African highland bananas used in this study in comparison with the 7 dessert (AAA) bananas, 5 Asian cooking bananas (ABB) and 15 East African highland bananas belonging to 5 clone sets, compared using 10 monomorphic descriptors in female fertile East African highland bananas. *ACB* Asian Cooking Bananas; *DB* Dessert Bananas; *EAHB* East African Highland Bananas; *tsEAHB* this study East African Highland Bananas; *Mbi* Mbidde; *Mus* Musakala; *Naka* Nakabululu; *Naki* Nakitembe; *Nfu* Nfuuka

The cladogram (Fig. 1) grouped East African highland banana cultivars close to each other. Cultivars in the Nakabululu clone set, the Nfuuka clone set and the Mbidde clone set formed the major cluster while cultivars in the Musakala clone set and the Nakitembe clone set (except cultivar Mbwazirume) formed a minor cluster next to the main cluster for the EAHB. The Asian cooking bananas did not cluster together, neither did the dessert (AAA) cultivars.

## Discussion

A good morphological descriptor should be stable, heritable, distinctly identifiable, easy to distinguish by the human eye, expressed consistently and able to clearly distinguish the individuals of interest. The high variation exhibited by the quantitative descriptors: fruit length, number of hands per bunch and number of fruits on the mid hand of the bunch, is an indication that such descriptors are not stable and thus not suitable for description of the EAHB cultivars. Javed et al. (2002) characterized 16 populations of Malaysian wild *M. acuminata* using 46 morphological characters and also found out that the quantitative characters were unstable. However in their study, they found pseudostem colour, petiole sheath colour and rachis position as useful characters to distinguish the *M. acuminata* populations, which is contrary to the findings in this study.

Each cultivar had a set of descriptors that were stable between the individuals of that cultivar but these descriptors were not useful for distinguishing a particular cultivar because in most cases the same descriptor was shared with two or more other cultivars. The 10 descriptors that were stable across the 11 cultivars, had the same mode score across the cultivars. For example, sap colour had a mode of 2 representing milky sap, edge of petiole margin had a mode of 2 representing red-purple color or brown when dried, colour of cigar leaf dorsal surface had a mode of 3 representing medium green color, bract behaviour before falling had a mode of 1 representing revolute (rolling), lobe colour of compound tepal had a mode of 2 representing yellow color, bract imbrication had a mode of 1 representing old bracts overlap at apex of bud (no imbrication), compound tepal basic colour had a mode of 2 representing cream color, anther colour had a mode of 6 representing pink/pink-purple, dominant colour of male flower had a mode of 2 representing cream, and fruit shape had a mode of 1 representing straight or slightly curved. This implies that these stable descriptors are not suitable for discriminating between the EAHB cultivars. However, they can be used to distinguish the East African highland bananas as a group from other groups of bananas. Therefore, there is a need to revise the available minimum set of *Musa* morphological descriptor to find suitable ones capable of distinguishing EAHB cultivars. Kitavi et al. (2016) and Christelová et al. (2016) studied the genepool of the triploid East African highland bananas using SSR and AFLP markers. They found that EAHB cultivars were genetically uniform. However from our study, the results from morphological characterization do not agree with the molecular findings since the EAHB cultivars used in this study expressed stable and consistent similar behaviour in only 10 characters out of the 31 characters, representing only 32% level of similarity. There is therefore a need to study the genetic basis of the morphological variation in EAHB cultivars using high-density genotyping by sequencing.

The fact that all descriptors with *P* values having ‘***’, ‘**’ and ‘*’ levels of significance (Table 4) corresponded to lower bound values higher than 0.5 (50%) (Table 5), while the descriptors with *P* values showing ‘NS’ had lower bound values less than 0.5 (50%) is a confirmation that all the stable descriptors had more than 50% mode score within a cultivar. This is in agreement with the tested hypotheses; the null hypothesis being that “the probability of getting a mode score is equal to the probability of getting a non mode score (*P* = 0.5)”, versus the alternative hypothesis “the probability of getting a mode score is greater than 0.5 (*P* > 0.5)”. Accordingly, if the null hypothesis is true, the descriptor is unstable, whereas if the alternative hypothesis is true, the descriptor is stable.

In order to minimize sources of variation during characterization and to have consistency in scoring, the gene bank curator or a specified team should be responsible for measuring and recording the descriptors. However, the number of individuals sampled also influenced the lower bound in that cultivars with low numbers of individuals sampled showed lower bounds for descriptors much lower than those cultivars with higher number of individuals sampled.

The light green margins with purple stripes on the bract external face and the yellow or green bract internal face that turns gradually to orange-red towards the apex, are characters which can be used to distinguish cultivar ‘Tereza’ from other EAHB (Online Resource 1).

The cladogram (Fig. 1) grouped EAHB close to each other. Cultivars in the Nakabululu clone set, the Nfuuka clone set and the Mbidde clone set formed a major cluster while cultivars in the Musakala clone set and the Nakitembe clone set (except cultivar Mbwazirume) formed a minor cluster next to the main cluster for the EAHB. This is in agreement with the observation by Karamura et al. (2016) who used SSR markers to assess the genetic variation within and between 53 banana groups. They found that the genetic distance was shortest within ilalyi and EAHB. However, within the EAHB, the variation was higher in the Nakitembe and Musakala clone sets. This was attributed to the fact that Nakitembe and Musakala are the clone sets containing most of the commercial cultivars, and the variation may be due to high and long-term selection pressure. The Asian cooking bananas (ABB) did not cluster together, neither did the dessert (AAA) cultivars. This may be because the set of descriptors used are neither suitable for grouping Asian cooking bananas nor dessert cultivars. Another reason might be that some of the selected descriptors’ data were missing for some cultivars in *Musalogue*. Hence, the *Musalogue* needs to be regularly updated to fill in the missing information about *Musa* germplasm. Grande Naine, Williams and Red Dacca clustered close to the EAHB cultivars, possibly because they are all triploid AAA cultivars and more closely related to the EAHB.

Molecular markers have been used in assessing the variation and relationships within and among different banana groups. Ortiz and Swennen (2014) indicated that DNA markers can be used as a tool to facilitate taxonomy and assessment of cultivar trueness-to-type. They referred to new microsatellites as being widely used for assessing diversity in bananas, plantains and other related crop wild relatives, some of which derived from expressed sequenced tags (EST) or from genomic sequence surveys (GSS). For example, Christelová et al. (2016) used simple sequence repeats (SSR) markers to characterize the global *Musa* germplasm collection kept at the international Transit Centre (ITC) in Leuven (Belgium). They found out that SSR marker assessment for 84% of the ITC accessions analyzed, agreed with the previous morphologically based classification while for 16% of the ITC accessions it did not. However, Creste et al. (2003), using SSR to analyze 35 polyploid banana cultivars (3*x* AAA, AAB; 4*x* AAAA, AAAB) grown in Brazil, concluded that their phenetic analysis based on the Jaccard similarity index highly agreed with the morphological classification. Kitavi et al. (2016) used 100 SSR markers to investigate the genetic diversity of 90 phenotypically diverse EAHB cultivars collected from Kenya and Uganda and compared them with plantain (AAB) and dessert (AAA) cultivars. They found out that EAHB cultivars had minimal genetic variation and were largely genetically uniform, irrespective of source of collection. They observed no association between EAHB genetic diversity classification according to SSR markers and morphological based classification for EAHB germplasm.

## Conclusion

In summary, this research shows that the minimum set of descriptors developed for banana consists of stable (32%) and unstable descriptors and is inefficient to differentiate between cultivars like in a small sample of the ‘matooke’ banana cultigen. The available set of minimum morphological descriptors in *Musa* should be revised to include only those that are stable and which can efficiently distinguish the East African Highland bananas. Likewise, a minimum set of high-throughput dense DNA markers should be defined for an improved assessment of diversity in *Musa* germplasm (Nunes de Jesus et al. 2009), which will complement the morphological characterization. A similar kind of research should be initiated on all *Musa* subgroups like the morphological diverse sub group of plantain, to find out whether the minimum set of descriptors is useful or not (De Langhe et al. 2005).

## Notes

**Open Access** This article is distributed under the terms of the Creative Commons Attribution 4.0 International License (http://creativecommons.org/licenses/by/4.0/), which permits unrestricted use, distribution, and reproduction in any medium, provided you give appropriate credit to the original author(s) and the source, provide a link to the Creative Commons license, and indicate if changes were made.
