# Supplementary material for: Suitability of existing Musa morphological descriptors to characterize East African highland ‘matooke’ bananas
Source: Genet Resour Crop Evol. 2017 Sep 18;65:645–57. doi: 10.1007/s10722-017-0562-9 (PMC7705172; doi:10.1007/s10722-017-0562-9)
Supplement: Supplementary file 1 [file GRCE-65-645-s001.docx]

| 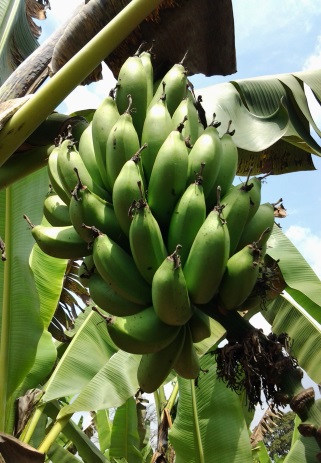 | | 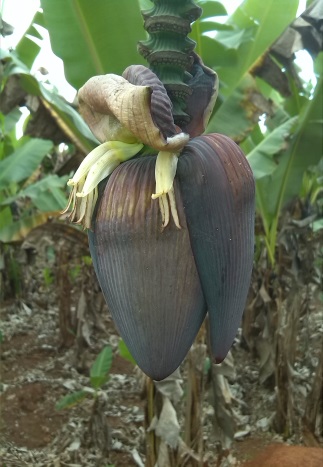 | | 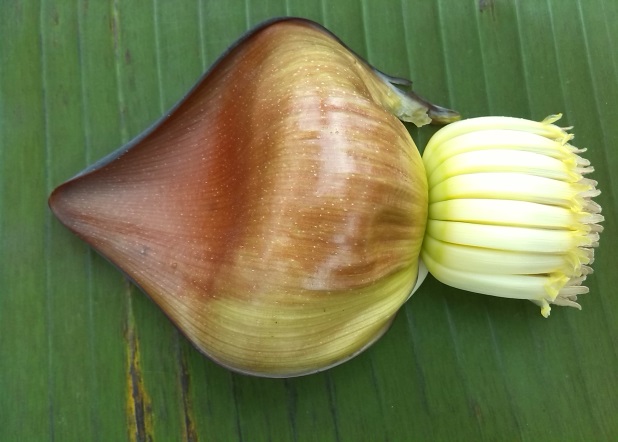 | |  |
| --- | --- | --- | --- | --- | --- | --- |
| **Kazirakwe** bunch | | Purple-brown bract external face | | Orange-red colour of bract internal face and cream compound tepal | |  |
| 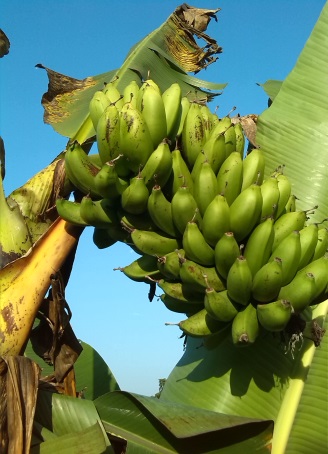 | | 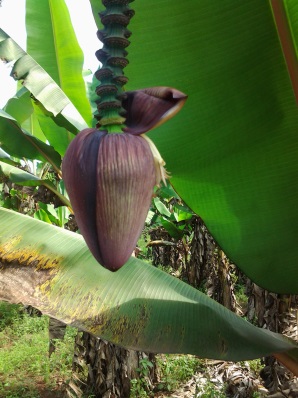 | | 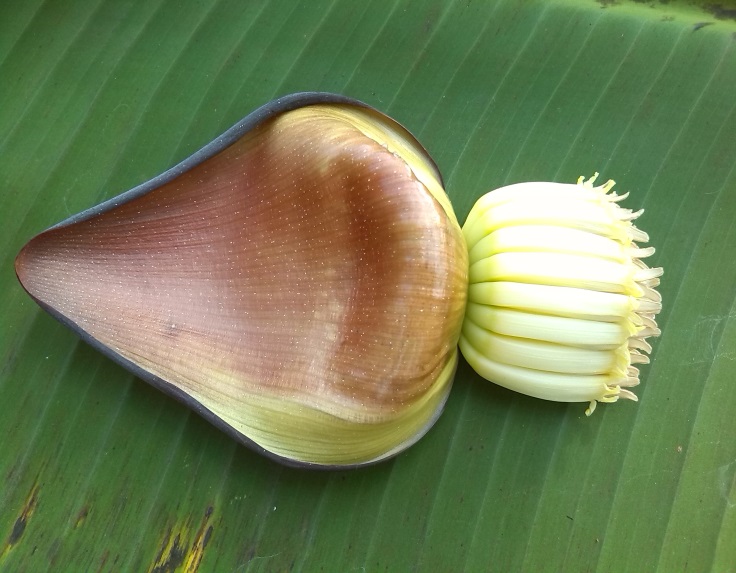 | |  |
| **Nakasabira** bunch | | Purple-brown bract external face | | Orange-red bract internal face and cream compound tepal | |  |
|  |  | |  | | | |
| 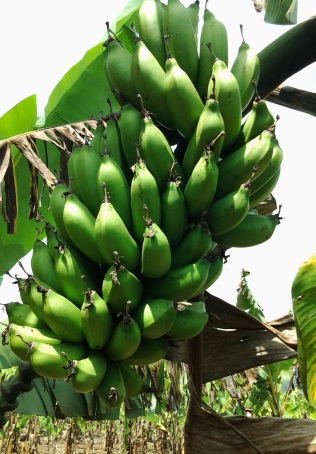 | 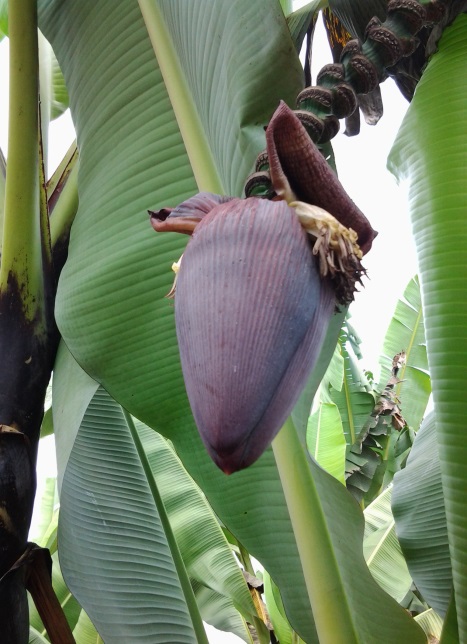 | | 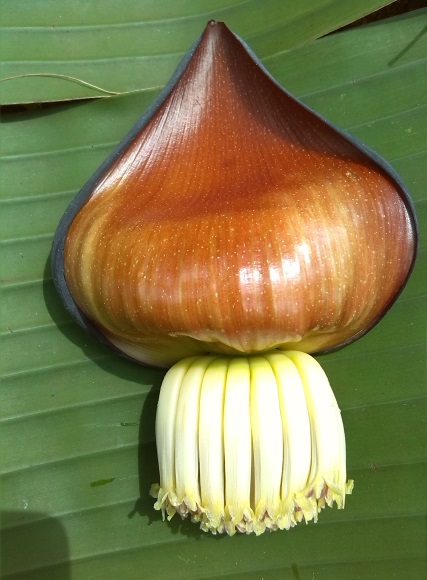 | |  | |
| **Nakayonga** bunch | Purple-brown bract external face | | Orange-red bract internal face and cream compound tepal | | | |
| 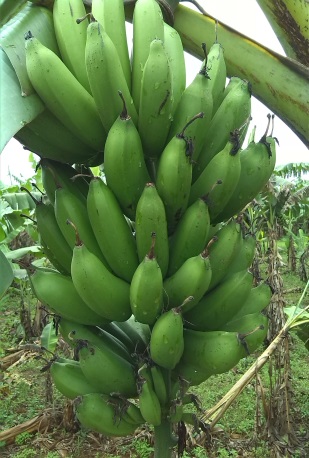 | 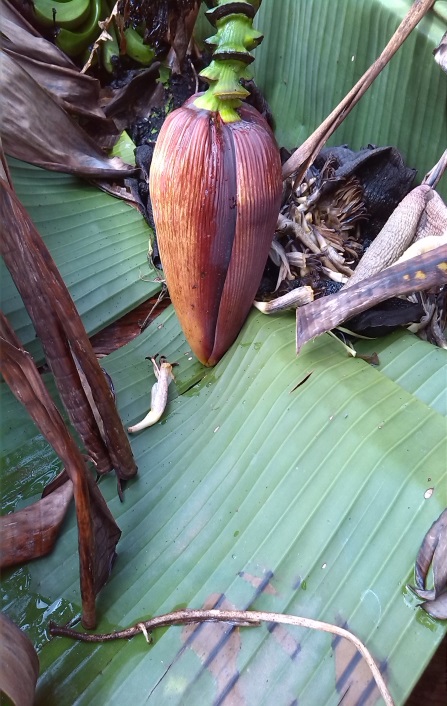 | | 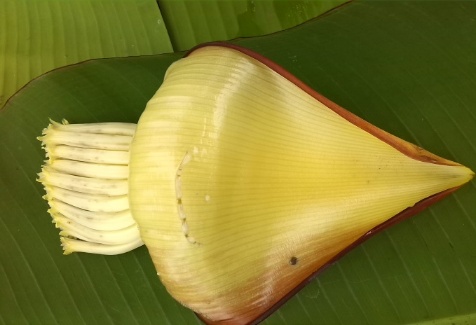 | |  | |
| **Nakyetengu bunch** | Purple brown bract external face | | Orange-red bract internal face and cream compound tepal | | | |
| 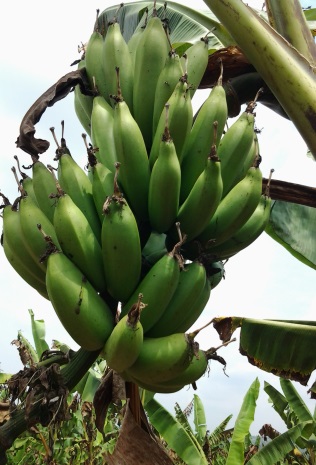 | 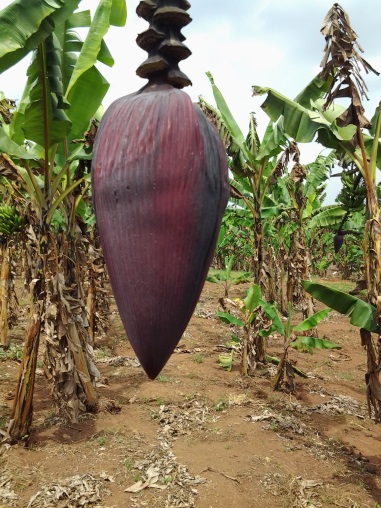 | | 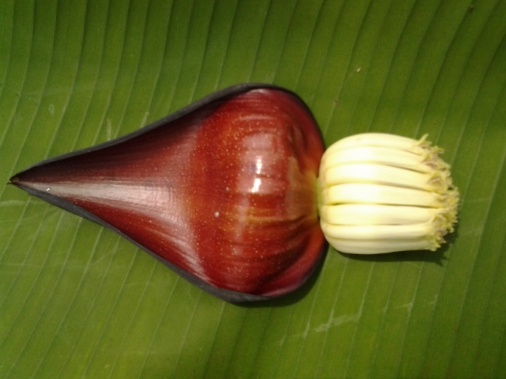 | |  | |
| **Entukura** bunch | Red-purple bract external face | | Red bract internal face and cream compound tepal | | | |
| 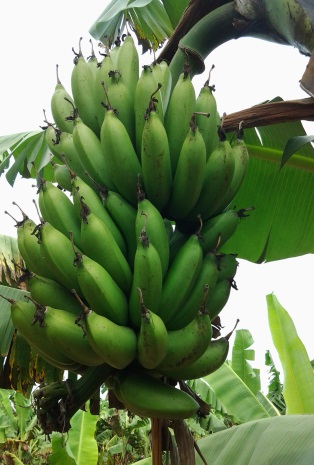 | 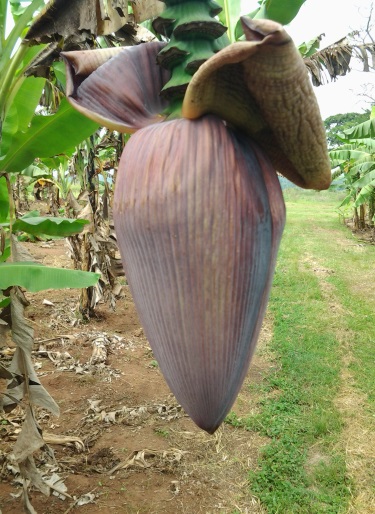 | | 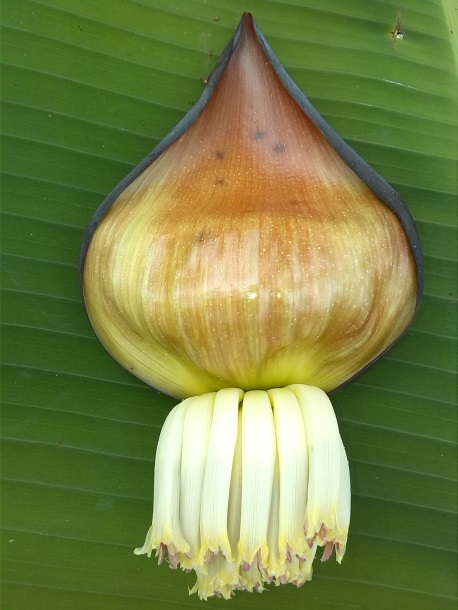 | |  | |
| **Enyeru** bunch | Purple-brown external bract face | | Orange-red bract internal face and cream compound tepal | | | |
| 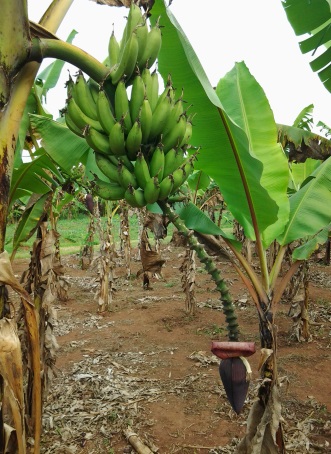 | 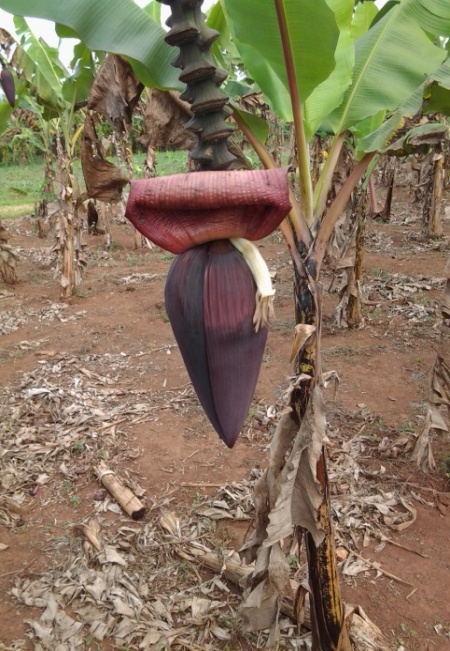 | | 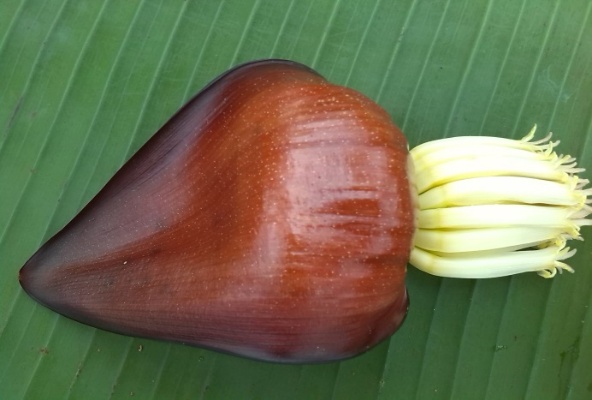 | |  | |
| **Enzirabahima** bunch | Red purple external bract face | | Red internal bract face and cream compound tepal | | | |
| 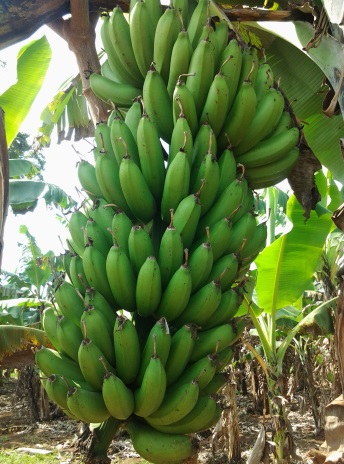 | 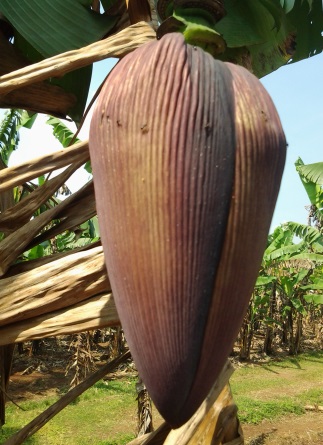 | | 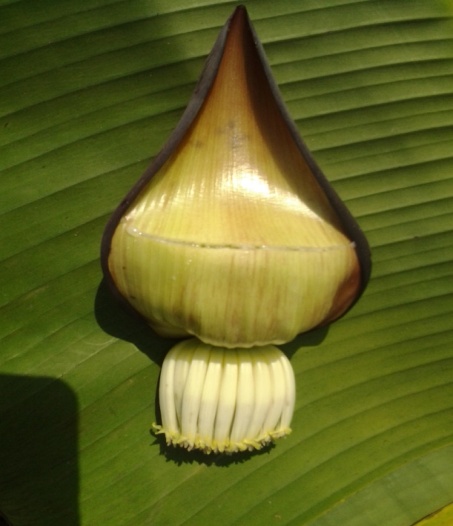 | |  | |
| **Kabucuragye** bunch | Purple-brown bract external face | | Orange-red bract internal face and cream compound tepal | |  | |
| 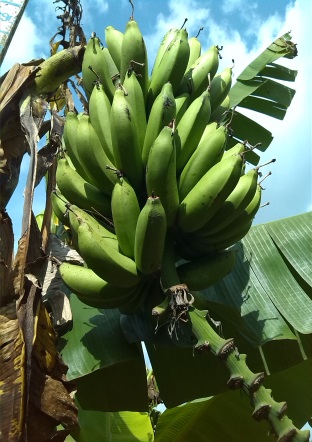 | 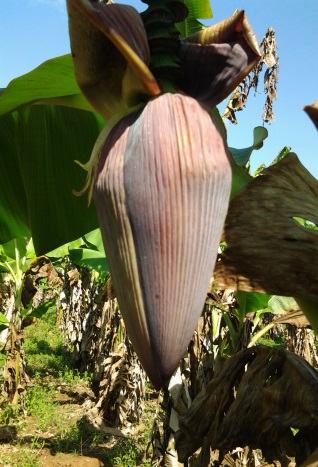 | | 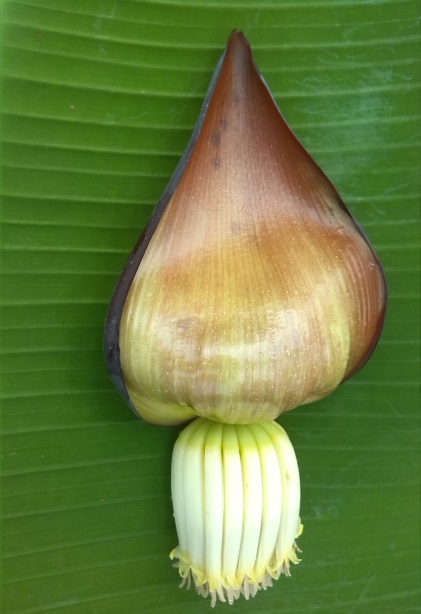 | |  | |
| **Namwezi** bunch | Purple-brown bract external face | | Orange-red bract internal face and cream compound tepal | | | |

| 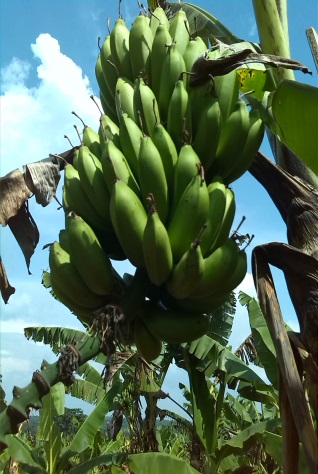 | 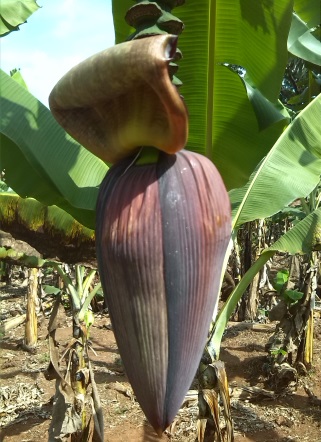 | 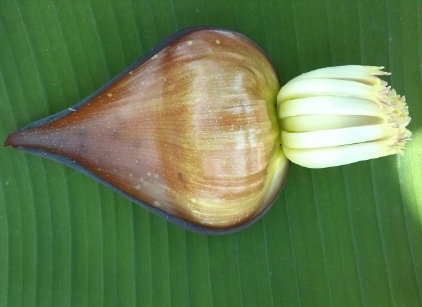 |  |
| --- | --- | --- | --- |
| **Nfuuka** bunch | purple-brown bract external face | Orange-red bract internal face and cream compound tepal | |

| 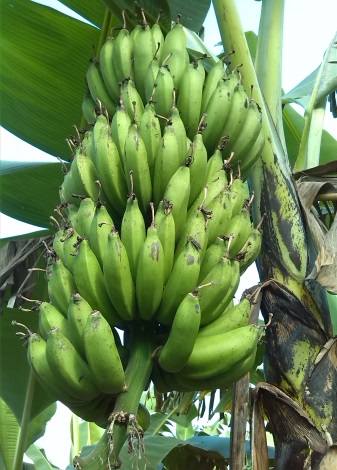 | 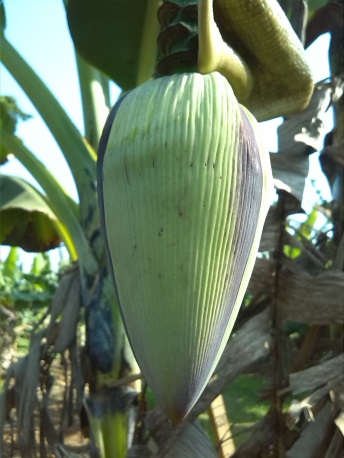 | 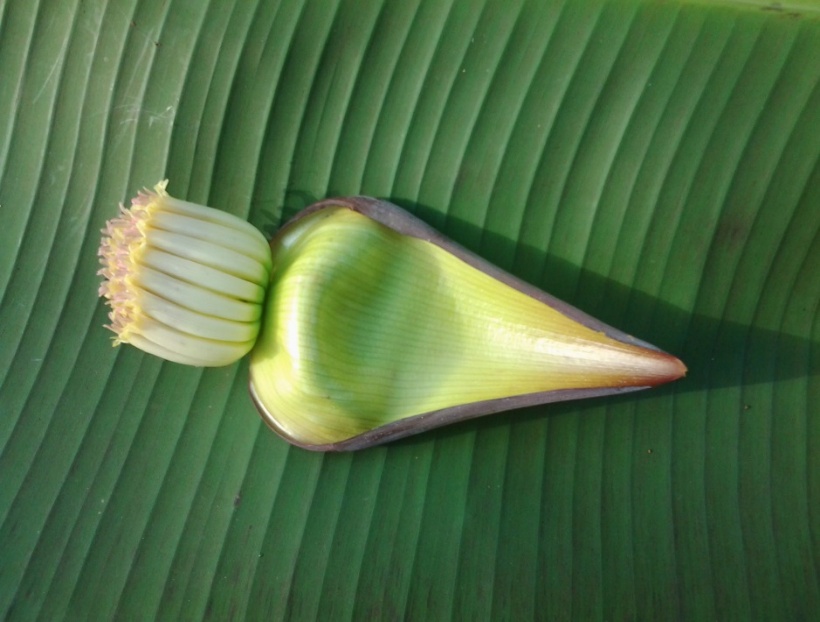 |
| --- | --- | --- |
| **Tereza** bunch | Light green with purple stripes of bract external face | Yellow or green with orange-red towards the apex of bract internal face |

**Fig.S1** Pictures of the eleven ‘matooke’ cultivars used in verifying the minimum set of *Musa* descriptors (Online Resource 1)
